# A Comprehensive Guide to Artificial Intelligence in Endoscopic Ultrasound

**DOI:** 10.3390/jcm12113757

**Published:** 2023-05-30

**Authors:** Kareem Khalaf, Maria Terrin, Manol Jovani, Tommy Rizkala, Marco Spadaccini, Katarzyna M. Pawlak, Matteo Colombo, Marta Andreozzi, Alessandro Fugazza, Antonio Facciorusso, Fabio Grizzi, Cesare Hassan, Alessandro Repici, Silvia Carrara

**Affiliations:** 1 Division of Gastroenterology, St. Michael’s Hospital, University of Toronto, Toronto, ON M5S 1A1, Canada; kareem.khalaf@mail.utoronto.ca (K.K.); pawlakatarzyna@gmail.com (K.M.P.); 2 Division of Gastroenterology and Digestive Endoscopy, Humanitas Research Hospital IRCCS, Rozzano, 20089 Milan, Italy; maria.terrin@humanitas.it (M.T.); marcospadaccini9@gmail.com (M.S.); matteo.colombo@humanitas.it (M.C.); marta.snpp@gmail.com (M.A.); alessandro.fugazza@humanitas.it (A.F.); cesareh@hotmail.com (C.H.); alessandro.repici@hunimed.eu (A.R.); 3 Division of Gastroenterology, Maimonides Medical Center, SUNY Downstate University, Brooklyn, NY 11219, USA; maj082@mail.harvard.edu; 4 Department of Biomedical Sciences, Humanitas University, Pieve Emanuele, 20089 Milan, Italy; tommy.rizkala@st.hunimed.eu; 5 Section of Gastroenterology, Department of Medical and Surgical Sciences, University of Foggia, 71122 Foggia, Italy; antonio.facciorusso@unifg.it; 6 Department of Immunology and Inflammation, Humanitas Research Hospital IRCCS, Rozzano, 20089 Milan, Italy; fabio.grizzi@humanitasresearch.it

**Keywords:** endoscopic ultrasound, artificial intelligence, biopsy, pathological diagnosis

## Abstract

Background: Endoscopic Ultrasound (EUS) is widely used for the diagnosis of bilio-pancreatic and gastrointestinal (GI) tract diseases, for the evaluation of subepithelial lesions, and for sampling of lymph nodes and solid masses located next to the GI tract. The role of Artificial Intelligence in healthcare in growing. This review aimed to provide an overview of the current state of AI in EUS from imaging to pathological diagnosis and training. Methods: AI algorithms can assist in lesion detection and characterization in EUS by analyzing EUS images and identifying suspicious areas that may require further clinical evaluation or biopsy sampling. Deep learning techniques, such as convolutional neural networks (CNNs), have shown great potential for tumor identification and subepithelial lesion (SEL) evaluation by extracting important features from EUS images and using them to classify or segment the images. Results: AI models with new features can increase the accuracy of diagnoses, provide faster diagnoses, identify subtle differences in disease presentation that may be missed by human eyes, and provide more information and insights into disease pathology. Conclusions: The integration of AI in EUS images and biopsies has the potential to improve the diagnostic accuracy, leading to better patient outcomes and to a reduction in repeated procedures in case of non-diagnostic biopsies.

## 1. Introduction

Endoscopic ultrasound (EUS) has revolutionized the field of gastrointestinal (GI) endoscopy by providing high-resolution imaging of the gastrointestinal tract and adjacent anatomical structures. EUS has been widely used for the diagnosis of bilio-pancreatic diseases, staging of GI tract tumors, evaluation of subepithelial lesions, and sampling of lymph nodes and solid masses [1]. EUS-guided fine-needle aspiration (FNA) and biopsy (FNB) have enabled the diagnosis of various malignancies and have greatly improved patient outcomes [2].

However, the accuracy of EUS-guided FNA and FNB largely depends on the skills and experience of the endoscopist and of the pathologist. In recent years, artificial intelligence (AI) has emerged as a promising tool for improving the accuracy and efficiency of EUS-guided tissue sampling and pathological diagnosis [3].

AI refers to the use of computer algorithms to analyze large amounts of data and identify patterns or make predictions. In healthcare, AI has been applied to various tasks, including image recognition, natural language processing, and clinical decision-making [4]. AI algorithms can analyze EUS images and assist with the interpretation of findings, as well as predict the pathological diagnosis of tissue samples obtained by EUS-guided FNA and FNB.

The objective of this review article is to provide an overview of the current state of AI in EUS imaging and pathology on the final pathological diagnosis. We will discuss the various AI techniques used for EUS image analysis and pathological diagnosis, their strengths and limitations, and their potential impact on clinical practice. A section is dedicated to the application of AI in training program to improve the knowledge of EUS and the recognition of anatomical structures. 

## 2. AI Algorithms and Image Acquisition

The use of artificial intelligence in EUS image interpretation has shown great potential for improving the accuracy and efficiency of the diagnostic process. AI algorithms can be divided into two main categories: deep learning techniques and machine learning techniques.

**Figure 1:**
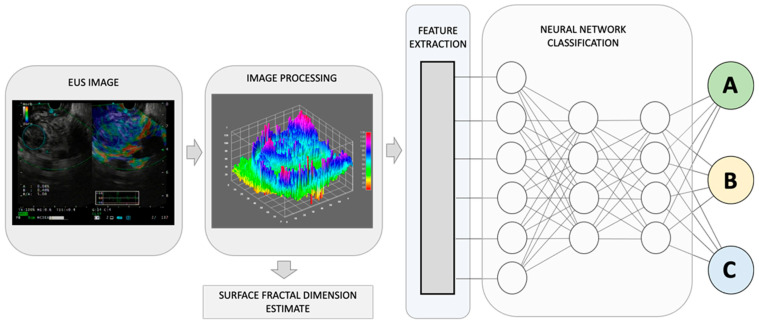
By combining recognized EUS-image features for pancreatic lesion diagnosis with measurements of non-Euclidean anatomical features, significant progress can be made in distinguishing diverse sub-types that have varying outcomes, A, B, and C. The utilization of fractal geometry, specifically the surface fractal dimension, as a measure of the space-filling property of an irregularly shaped structure, can be effectively merged as a feature within an AI-based neuronal network classification system, to achieve a more precise anatomical classifier system.

Deep learning techniques involve the use of neural networks to learn and recognize patterns in EUS images (Figure 1). These networks are composed of multiple layers of interconnected nodes that allow for the processing of large amounts of data. Convolutional neural networks (CNNs) are a commonly used deep learning technique for image analysis in healthcare [5]. They are designed to identify and extract important features from EUS images and use them to classify or segment the images.

Machine learning techniques, on the other hand, involve the use of algorithms that can learn from data and make predictions based on that learning. These techniques can be supervised, unsupervised, or semi-supervised. Supervised learning involves the use of labelled data to train an algorithm to recognize patterns in EUS images. Unsupervised learning involves the use of unlabeled data to discover patterns and relationships in the data. Semi-supervised learning combines both supervised and unsupervised learning [6]. In addition to image interpretation, AI can also be used to enhance the acquisition of EUS images. This includes automatic segmentation and image quality improvement.

Automatic segmentation involves the use of AI algorithms to identify and separate different structures in EUS images [7]. This can help to improve the accuracy and efficiency of EUS-guided procedures by providing better visualization of the target area. For example, AI algorithms can be used to automatically segment the pancreas or the lymph nodes in EUS images, allowing for more precise targeting during EUS-guided biopsy [8]. 

Image quality improvement involves the use of AI algorithms to enhance the clarity and resolution of EUS images. This can help to improve the accuracy of image interpretation and diagnosis. For example, AI algorithms can be used to reduce noise, improve contrast, and sharpen edges in EUS images. AI-enhanced image quality can also help to reduce the variability in image quality between different endoscopists and ultrasound machines, improving the consistency of diagnosis and treatment [9].

## 3. Lesion Detection and Characterization

### 3.1. Tumor Identification

Tumor identification is a crucial step in the diagnosis and staging of GI neoplasia, especially in case of pancreatic cancers that may be isoechoic with the surrounding parenchyma or may be hidden by signs of chronic pancreatitis. AI algorithms can assist with tumor identification by analyzing EUS images and identifying suspicious areas that may require biopsy sampling and microscopy observation or further clinical evaluation [10]. Deep learning techniques, such as CNNs, have shown great potential for tumor identification by extracting important features from EUS images and using them to classify or segment the images [11]. Supervised machine learning techniques can also be used to train AI algorithms to recognize specific tumor features, such as shape, size, and vascularity [12,13]. A recent meta-analysis of 10 studies, which involved 1871 patients, evaluated the diagnostic accuracy of AI applied to EUS in detecting pancreatic cancer (Table 1). The results showed that AI had a high diagnostic sensitivity of 0.92 and specificity of 0.9, with an area under the summary receiver operating characteristics (SROC) curve of 0.95 and a diagnostic odds ratio of 128.9 [14]. These findings suggest that AI-assisted EUS could become an essential tool for the computer-aided diagnosis of pancreatic cancer. However, a relatively small number of studies and enrolled patients make generalizing difficult, and further research is needed to validate these results on a larger scale.

It is known that there are several advantages of incorporating new features in classifying pathological changes using AI. Among these are the following: (a) increased accuracy: the addition of new features to AI models can increase their accuracy in diagnosing and classifying pathological changes; (b) faster diagnosis: AI models with new features can analyze large amounts of data quickly and accurately, allowing for early diagnosis and better patient outcomes with reduced healthcare costs; (c) personalized treatment: AI models with new features can identify subtle differences in disease presentation that may be missed by human eyes. This can lead to more personalized treatment plans that are tailored to the specific needs of each patient; (d) improved decision-making: AI models with new features can provide clinicians with more information and insights into disease pathology, allowing them to make more informed decisions about patient care; and (e) scalability: AI models can be easily scaled to analyze large amounts of data, making them ideal for analyzing large datasets or monitoring patient health over time. This can lead to improved population health management and disease surveillance. 

Diseases of various origins, such as inflammatory disorders, tumors, and functional diseases, can result in changes in the structural complexity and dynamic activity patterns. One way to quantify this structural complexity is by measuring the fractal dimension, among other parameters. 

The human body is composed of intricate systems and networks, including its most complex structures. It is now widely accepted that the architecture of anatomical entities and their activities exhibit non-Euclidean properties. Natural fractals, including those found in anatomy, possess four distinct characteristics: (a) irregular shape, (b) statistical self-similarity, (c) non-integer or fractal dimension, and (d) scaling properties that depend on the scale of measurement. As anatomical structures do not conform to regular Euclidean shapes, their dimensions are expressed as non-integer values between two integer topological dimensions [17]. Fractal geometry has been shown to be useful in evaluating the geometric complexity of anatomic and imaging patterns observed in both benign and malignant masses (Figure 1).

Recently, Carrara et al. have introduced a new estimator, called the surface fractal dimension, to evaluate the complexity of EUS-Elastography images in differentiating solid pancreatic lesions [18]. The study showed that the surface fractal dimension can distinguish malignant tumors from NETs, unaffected tissues surrounding malignant tumors from NETs, and NETs from inflammatory lesions. This study highlights the importance of incorporating fractal analysis into AI algorithms for the diagnosis and categorization of the diverse array of pancreatic lesions.

### 3.2. Subepithelial Lesion Evaluation

Subepithelial lesions (SELs) are a common indication to perform EUS, and their diagnosis can be challenging. AI algorithms can assist with SELs evaluation by analyzing EUS images and identifying suspicious lesions that may require biopsy. Deep learning techniques, such as CNNs, have shown great potential for SELs evaluation by extracting important features from EUS images and using them to classify or segment the images. The study by Hirai et al. suggests that an AI system has higher diagnostic performance than experts in differentiating SELs on EUS images [19]. The AI system’s accuracy for classifying five different types of SELs was 86.1%, which was significantly better than that of all endoscopists. In particular, the sensitivity and accuracy of the AI system for detecting gastrointestinal stromal tumors (GISTs) were higher than those of all endoscopists. These findings suggest that AI technology can be a valuable tool to assist in the diagnosis of SELs on EUS images, and may help improve clinical decision-making [19].

### 3.3. Diagnostic Accuracy

The diagnostic accuracy of AI algorithms may be affected by several factors, such as the quality of the EUS images, the size and location of the lesion, and the expertise of the endoscopists and the pathologists. The results of previous studies have been controversial. In recent meta-analysis, Xiao et al. identified seven studies to assess the diagnostic accuracy of AI-based EUS in distinguishing GISTs from other SELs [20]. The combined sensitivity and specificity of AI-based EUS were 0.93 and 0.78, respectively, with an overall diagnostic odds ratio of 36.74 and an area under the summary receiver operating characteristic curve (AUROC) of 0.94. These results suggest that AI-based EUS showed high diagnostic ability in differentiating GISTs from other SELs and could potentially set a premise for adapting diagnostic capabilities of other disease under EUS.

### 3.4. Clinical Impact and Limitations

The clinical impact of AI algorithms for lesion detection and characterization in EUS-guided pathological diagnosis is still under investigation. However, studies have reported that AI algorithms can improve the accuracy and efficiency of EUS-guided procedures, reduce the need for unnecessary biopsies, and assist with treatment planning [21]. AI algorithms can also help to reduce the inter-observer variability in lesion detection and characterization between different endoscopists [22]. However, the implementation of AI algorithms in clinical practice may be limited by several factors, such as the availability and cost of AI software, the need for specialized training, concerns about data privacy and security, and most importantly the need for larger studies to establish the accuracy of such systems.

## 4. Digital Histopathological Diagnosis

The advancement of digital pathology has revolutionized the field of pathology by enabling the acquisition, management, and interpretation of pathological information in a digital format. This transition has been fueled by advances in whole slide imaging (WSI) technology, which allows for the digitization of glass slides at high resolution [23]. The adoption of digital pathology offers numerous advantages, such as improved efficiency, reduced turnaround times, remote consultation, and easy access to archived cases [23]. The ability to store pictures from tissue acquisition, as it happens for radiological imaging, puts the basis to share and use a lot of knowledge from pathological anatomy. Moreover, it sets the stage for the application of AI algorithms to facilitate and enhance diagnostic accuracy in the field of EUS.

WSI involves the scanning of entire histological glass slides to create high-resolution digital images. These digital images can be zoomed in or out and navigated as easily as a glass slide under a microscope. WSI technology has been instrumental in overcoming the challenges of data management in digital pathology, as it allows for efficient storage, retrieval, and sharing of massive amounts of image data [23]. Furthermore, WSI facilitates the standardization of image quality and provides an ideal platform for the application of AI algorithms to analyze the digital images, thereby supporting the development of novel diagnostic tools in endoscopic ultrasound.

In the context of histopathological image analysis, CNNs can be trained to automatically detect and classify the multifarious tissue structures, cellular patterns, and pathological alterations. CNNs are composed of multiple layers of interconnected “neurons”, including convolutional, pooling, and fully connected layers. The hierarchical structure of CNNs allows them to learn complex, high-level features from raw image data, thereby making them particularly suitable for the analysis of intricate histopathological images in endoscopic ultrasound [24,25,26].

In addition to CNNs, other machine learning techniques have been employed in histopathological image analysis for endoscopic ultrasound. These include support vector machines (SVM), random forests, and decision trees, among others. These algorithms can be used to extract and analyze peculiar features from histopathological images, such as texture, shape, and color. By leveraging the strengths of multiple machine learning techniques, ensemble models can be created to improve overall performance and address potential limitations of individual algorithms [27].

The integration of AI algorithms, particularly CNNs, into the field of EUS has the potential to revolutionize the diagnosis and management of various GI tract disorders. As research progresses and these techniques become more refined, AI-based tools are expected to play an increasingly prominent role in the field of EUS and digital pathology.

## 5. AI in Pathological Diagnosis

### 5.1. AI Applications in Anatomical Pathology

Tumor grading and staging are critical steps in the management of GI malignancies. Accurate tumor grading and staging are necessary for determining the appropriate treatment plan and predicting patient outcomes. AI algorithms can aid in tumor grading and staging by analyzing EUS images and identifying features that correspond to different tumor stages and grades (Figure 1). Deep learning techniques, such as CNNs, can identify subtle differences in tissue structure and morphology that may not be apparent to the human eye. For example, CNNs can potentially analyze EUS images of pancreatic cancer and differentiate between early-stage and advanced-stage tumors based on changes in tissue texture and vascularity [15,16,28]. AI algorithms can also predict the presence of lymph node metastasis by analyzing EUS images and identifying characteristic features, such as size, shape, and echogenicity, (Table 2). In a study by Săftoiu et al., contrast-enhanced harmonic EUS (CEH-EUS) with time-intensity curve (TIC) analysis and artificial neural network (ANN) processing were used to differentiate pancreatic carcinoma (PC) and chronic pancreatitis (CP) cases [29]. Parameters obtained through TIC analysis were able to differentiate between PC and CP cases and showed good diagnostic results in an automated computer-aided diagnostic system.

Prognostic and predictive biomarker analysis is essential for predicting patient outcomes and determining the most appropriate treatment plan. Prognostic biomarkers are associated with patient outcomes, such as survival or recurrence, while predictive biomarkers are associated with response to specific therapies [30]. Kurita et al. investigated the diagnostic ability of carcinoembryonic antigen (CEA), cytology, and AI using cyst fluid in differentiating malignant from benign pancreatic cystic lesions [31]. AI using deep learning showed higher sensitivity and accuracy in differentiating malignant from benign pancreatic cystic lesions than CEA and cytology. 

### 5.2. Integrating AI in EUS-Guided Tissue Acquisition

EUS-guided fine needle aspiration (EUS-FNA) and EUS-guided fine needle biopsy (EUS-FNB) are commonly used techniques for obtaining tissue samples for pathological diagnosis. The accuracy of EUS-guided tissue acquisition largely depends on the skills and experience of the operator and the quality and size of the tissue samples obtained can vary. The integration of AI in EUS-guided tissue acquisition has the potential to improve the accuracy and efficiency of the procedure, leading to better patient outcomes (Table 3).

In a study by Inoue et al., an automatic visual inspection method based on supervised machine learning was proposed to assist rapid on-site evaluation (ROSE) for endoscopic ultrasound-guided fine needle aspiration (EUS-FNA) biopsy. The proposed method was effective in assisting on-site visual inspection of cellular tissue in ROSE for EUS-FNA, indicating highly probable areas including tumor cells [33].

Hashimoto et al. evaluated the diagnostic performance of their computer-aided diagnosis system using deep learning in EUS-FNA cytology of pancreatic ductal adenocarcinoma. The deep learning system showed promising results in improving diagnostic performance by step-by-step learning, with higher training volume and more efficient system development required for optimal CAD performance in ROSE of EUS-FNA cytology [32].

Ishikawa et al. developed a new AI-based method for evaluating EUS-FNB specimens in pancreatic diseases using deep learning and contrastive learning. The AI-based evaluation method using contrastive learning was comparable to macroscopic on-site evaluation (MOSE) performed by EUS experts and can be a novel objective evaluation method for EUS-FNB [21].

AI algorithms can potentially assist in EUS-FNA and EUS-FNB by providing real-time feedback to the endoscopist during the procedure. AI algorithms can analyze EUS images in real-time and provide guidance on the optimal location and depth of the needle insertion, as well as feedback on the quality of the tissue sample obtained. AI algorithms can also assist in the selection of the appropriate needle size and type based on the characteristics of the target lesion, such as diameter and location. The expectations of the limit of how AI can help improve variability in an endoscopic procedure can be the forefront need to find applicability from these systems to aid the outcome of a procedure [3].

## 6. Clinical Validation of AI-Enhanced Pathological Diagnosis

The accuracy of AI-enhanced pathological diagnosis has not been evaluated to a certain degree in the EUS setting. Traditional pathology approaches have been crucial in diagnosing diseases. Additionally, several qualitative and semi-quantitative grading and staging scoring systems have been widely proposed with even more accepted limitations. Semi-quantitative scores are not “measures” but only “labels” of severity [34]. An observer assigns “semiquantitative” scores to tissue changes based on predefined morphologic criteria. These scores are whole numbers and are less precise than quantitative scores because they only approximate relative changes. However, the advantage of semiquantitative scoring is that it can be applied to both macroscopic and microscopic tissue changes, generating strong data that can be statistically analyzed and used to evaluate experimental groups [35].

However, the development of digital pathology and AI solutions have allowed for more quantitative pathologic assessments, which are particularly useful in translational research [36]. These approaches provide invaluable opportunities for biomarker discovery and patient selection, aiding in the identification of optimal treatment regimens based on patient profiles [37]. Despite these benefits, challenges still exist in implementing AI-based methods in clinical settings. Specifically, in endoscopic setting, the incorporation of datasets and patient profiles to enhance the pathological diagnosis of a set disease is an expectation of many clinicians with the ever-growing boom of AI into clinical practice, that does not exist as of today.

The clinical impact of AI-enhanced pathological diagnosis has yet to be fully realized. However, the potential benefits include improved diagnostic accuracy, reduced inter-observer variability, and more efficient use of healthcare resources. AI-enhanced pathological diagnosis may also lead to the development of new biomarkers and treatment strategies for gastrointestinal malignancies. Limitations to the use of AI in pathological diagnosis exist, such as the accuracy of AI algorithms depends on the quality and quantity of the data used for training. The development of AI algorithms requires large amounts of data, which may be difficult to obtain for rare or uncommon gastrointestinal malignancies. Additionally, the use of AI in pathological diagnosis may raise ethical concerns regarding the role of technology in healthcare decision-making, a stage unexplored yet in many aspects of AI’s massive incorporation into healthcare. 

## 7. EUS Training

The learning of EUS requires time and practice in a high-volume center, with an experienced endosonographer as teacher. This is due to the need to learn not only endoscopy but also to show excellent knowledge of ultrasound anatomy and of different district diseases [38].

As in other areas, in EUS AI ideally aims to improve the quality of the examination, helping to distinguish the type of lesions found, reducing procedural times, and providing real-time decision support and guidance in the execution of operative procedures [39]. It could also support the training of beginners, speeding up the learning process and reducing the need for a mentor. Furthermore, AI could provide quality control, standardizing performance between trainees and experts (Table 4).

Often one of the first obstacles for the trainee in approaching EUS is the recognition of anatomical structures, as these are visualized in an unusual perspective, that varies according to the position of the endoscope and the station being examined. In 2021 at the ESGE days, a pilot study was awarded as the “best procedural innovation of the year”; it proposed an AI system based on two convolutional neuronal networks that recognizes anatomical structures in both radial and linear EUS. This software has already achieved a recognition accuracy of 85% during the development phase [43].

Similarly, another study proposed the use of a CNN consisting of two branches, one for voice data and one for image data. EUS image labels were assigned based on simple verbal inputs indicating anatomical landmarks provided by experienced operators during the procedures [42]. The prediction accuracy after the first system training reached 76% at the image level on a data set with five different labels. Moreover, voice tagging, instead of manual annotation, is very convenient in saving time [42]. These results are encouraging from the point of view of providing support to beginners, however data on actual improvement in the learning curve are scarce [42].

The BP MASTER (pancreaticobiliary master) system was specifically designed by a joint collaboration (by Renmin Hospital of Wuhan University, Wuhan Union Hospital of Huazhong University of Science and Technology, Wuhan Puai Hospital, and Wuhan EndoAngel Medical Technology Company) for training in EUS and examination quality control. The system includes a station classification model and a pancreas/abdominal aorta/portal confluence segmentation model. It was validated both internally and externally, reaching in the latter an accuracy of 82.4% in station classification and 0.72 Dice score in segmentation. The results of accuracy in classification and Dice score in segmentation were also comparable to that of experienced operators. In a crossover study, it was tested whether the system could increase the accuracy of station recognition in trainees, showing an improvement from 67.2% to 78.4% (*p* < 0.01) [45]. 

The same research group, subsequently, implemented the BP MASTER incorporating four deep convolutional neural networks (DCNN) in order to obtain additional functions: transducer location information, real-time operating instructions, and to annotate the common bile duct anatomy and measure its caliber on freeze frame [44]. At internal and external validations, the model confirmed its accuracy values, comparable to that of an expert.

Another crossover study was performed, aiming to evaluate the trainees’ accuracy improvement in interpreting the images when assisted by AI, which raised from 60.8% to 76.3% (*p* < 0.01) [44]. Finally, another study proposed to use AI to improve learning of the CH-EUS technique, particularly useful in identifying pancreatic masses and notoriously difficult to learn.

The system (CH-EUS MASTER), which includes a real-time acquisition and segmentation model, was adequately validated. A cross-trial was then conducted to assess the impact on trainees’ learning curve, using intersection over union (IoU) and time to lesion finding as indicators. Beginners who were supported by CH-EUS MASTER reported an improvement in mean IoU from 0.80 to 0.87 (*p* = 0.002) and a reduction in mean lesion identification times from 22.75 to 17.98 s (*p* < 0.01), and from 34.21 to 25.92 s (*p* < 0.01) in the pancreatic body-tail and head-uncinate process, respectively [41]. 

CH-EUS MASTER seems also a valid tool in guiding EUS-FNA, with improvement in the first-pass diagnostic yield [40]. The development of more effective and articulate AI systems is desirable to allow trainees to speed up the training process and improve their performance. Ideally, integrating AI assistance systems with the use of simulators, up to virtual reality, could almost make the mentor unnecessary, but further data are necessary [46,47]. 

## 8. Future Directions and Conclusions

The integration of AI in EUS images and biopsy microscopy analysis has the potential to improve the diagnostic accuracy, leading to better patient outcomes and to a reduction in repeated procedures in case of non-diagnostic biopsies. AI algorithms can also aid in tumor grading, staging, and prognostic analysis. However, the clinical impact of AI-enhanced pathological diagnosis has yet to be established. Further research is needed to evaluate the long-term benefits and limitations of AI in EUS-imaging and biopsies.

## Figures and Tables

**Table 1:** AI’s role in pancreatic cancer.

Study	Sinkala M et al., 2020, South Africa [8]	Wazir M. et al., 2019, USA [15]	Corral JE et al., 2019, USA [16]
AI type	ANN	ANN	ANN
Topic	differentiation of molecular-genomic profile of PDAC subtypes (mRNA and DNA methylation models)	PDAC risk prediction based on personal health data	IPMN characterization on MRI
Study population	45 pancreatic cancer samples	898 patients diagnosed with pancreatic cancer	139 patients with histologically characterized IPMNs (due to pancreatectomy) and previous MRI images
Main results	AI in differentiation of two PDAC subtypes: overall classification Ac 100% for the mRNA-based model, 99% for the DNA methylation-model; model provides predictions of clinical response to chemotherapy	AI Sn and Sp in testing cohort: 80.7%, 80.7%; AUROC curve 0.85.	AI in detect dysplasia Sn and Sp: 92%, 52%. Identification of high-grade dysplasia/cancer: Sn and Sp 75% and 78%. AI AUROC curves 0.78 (*p* = 0.90) vsAUROC base on AGA criteria 0.76, AUROC based on Fukuoka criteria 0.77.

Abbreviations: AI (Artificial Intelligence), PDAC (Pancreatic Ductal Adenocarcinoma), ANN (Artificial Neural Network), mRNA (Messenger RNA), DNA (Deoxyribonucleic Acid), IPMN (Intraductal Papillary Mucinous Neoplasm), MRI (Magnetic Resonance Imaging), Ac (Accuracy), Sn (Sensitivity), Sp (Specificity), AUROC (Area Under the Receiver Operating Characteristic Curve), and AGA (American Gastroenterological Association).

**Table 2:** Studies assessing diagnostic capabilities of AI systems in EUS.

Study	Hirai K. et al., 2022, Japan [19]	Marya NB. et al., 2021, USA [24]	Marya NB. et al., 2021, USA [25]	Oh CK. et al., 2021, South Korea [26]	Săftoiu A. et al., 2015, Denmark [29]	Zhang MM. et al., 2010, China [27]
AI type	CNN	CNN	CNN	CNN	ANN	SVM
Topic	differential diagnosis of SELs (five-category: GIST, leiomyoma, schwannoma, NET, ectopic pancreas)	enhance the diagnosis of AIP	identify and classify FLLs	differential diagnosis of SELs (GISTs and leiomyomas)	differential diagnosis of PDAC and CP using CH-EUS and TIC analysis	differential diagnosis of PDAC from normal tissue (based on 29 pattern features)
Study population	16,110 images, 631 examinations	583 patients, 1,174,461 still images from videos	256 patients, 210,685 still images from videos	114 patients (with histologically confirmed gastric GIST), 376 still images	167 patients with PDAC or CP	216 patients (153 with PDAC, 63 without)
Main results	overall Ac: AI 86.1% vs. expert endoscopy 68.0% (*p* < 0.001); Sn, Sp, Ac of AI in differentiating GISTs from non-GIST: 98.8%, 67.6%, 89.3% (better than expert endoscopist: Sn, Ac *p* < 0001)	AI processed 955 frames/sec. Sn and Sp for distinguishing AIP from PDAC: 90%, 93%. Sn and Sp for distinguishing AIP from all studied conditions (PDAC, CP, NP): 90%, 85%	AI autonomously locates FLLs in 92.0% of videos. Sn and Sp in classifying malignant FLLs on random still images: 90%, 71%. Sn and Sp in classifying malignant FLLs on full-length videos: 100% and 80%	AI Sn, Sp, Ac in per-image analysis: 95.6%, 82.1%, 91.2% Sn, Sp, Ac in per-patient analysis 100.0%, 85.7%, 96.3% (better than expert endoscopist: Sn, Ac *p* < 0001)	AI Sn Sp PPV and NPV using TIC analysis on CH-EUS: 94.64%, 94.44%, 97.24%, 89.47%	AI Ac, Sn, Sp, PPV and NPV for the diagnosis of pancreatic cancer: 97.98%, 94.32%, 99.45%, 98.65%, 97.77%

Abbreviations: AI (Artificial Intelligence), CNN (Convolutional Neural Network), ANN (Artificial Neural Network), SVM (Support Vector Machine), SELs (Subepithelial Lesions), GIST (Gastrointestinal Stromal Tumor), NET (Neuroendocrine Tumor), AIP (Autoimmune Pancreatitis), CP (Chronic Pancreatitis), CH-EUS (Contrast-Enhanced Endoscopic Ultrasound), TIC (Time-Intensity Curve), PDAC (Pancreatic Ductal Adenocarcinoma), FLLs (Focal Liver Lesions), NP (Normal Pancreas/Neuroendocrine Tumor), PPV (Positive Predictive Value), and NPV (Negative Predictive Value).

**Table 3:** Studies assessing AI’s pathological diagnosis.

Study	Ishikawa T. et al., 2022, Japan [21]	Kurita Y et al., 2019, Japan, [31]	Hashimoto Y. et al., 2018, Japan [32]	Inoue H. et al., 2014, Japan [33]
AI type	CNN	ANN	ANN	GMM
Topic	MOSE in pancreatic diseases	analysis of cyst fluid, cytology and EUS characteristics in differentiating malignant from benign pancreatic cysts	ROSE in PDAC	AI automatic visual inspection method is proposed to assist ROSE
Study population	96 patients, 173 specimens	85 patients (59 surgical specimens, 26 EUS-guided FNA specimens)	500 images of cytology specimen (stained and in high definition)	\
Main results	Initial study: AI Ac 71.8% (vs. MOSE performed by EUS experts 81.6%). Using contrastive learning: AI Sn, Sp, Ac: 90.34%, 53.5%, 84.39%, (vs. 88.97%, 53.5%, 83.24% of EUS experts)	AI diagnostic ability in malignant cystic lesions: AUROC curve 0.966 (vs. 0.719 for CEA, 0.739 for cytology) AI Sn, Sp, Ac: 95.7%, 91.9%, 92.9% (vs. CEA Sn 60.9%, *p* = 0.021; cytology Sn 47.8% *p* = 0.001; CEA Ac 71.8%, *p* < 0.00; cytology Ac 85.9%, *p* = 0.210)	AI Sn, Sp, Ac at the first learning stage: 78%, 60% 69% AI Sn, Sp, Ac at the second learning stage: 80%, 80%, 80%	The AI method is reported as helpful for EUS-FNA in aiding ROSE, indicating areas highly likely to include tumor cells

Abbreviations: AI (Artificial Intelligence), CNN (Convolutional Neural Network), ANN (Artificial Neural Network), GMM (Gaussian Mixture Model), MOSE (Magnifying Endoscopy with Narrow Band Imaging) in pancreatic diseases, ROSE (Rapid On-Site Evaluation), PDAC (Pancreatic Ductal Adenocarcinoma), EUS (Endoscopic Ultrasound), AUROC (Area Under the Receiver Operating Characteristic Curve), CEA (Carcinoembryonic Antigen), and Sn (Sensitivity), Sp (Specificity), and Ac (Accuracy).

**Table 4:** AI’s role in EUS training.

Study	Tang A. et al., 2023, China [40]	Tang A. et al., 2023, China [41]	Bonmati E. et al., 2022, UK [42]	Robles-Medranda C. et al., 2021, Ecuador [43]	Yao L. et al., 2021, China [44]	Iwasa Y. et al., 2021, Japan. [7]	Zhang J. et al., 2020, China [45]
AI type	CNN	CNN	CNN	CNN	CNN	CNN	CNN
Topic	pancreatic mass diagnosis with CH-EUS and AI guided EUS-FNA in real time (CH-EUS MASTER)	real-time capture and segmentation of solid pancreatic masses with CH-EUS (CH-EUS MASTER)	voice-assisted image labeling for AI image classification	real time recognition and characterization of anatomical structures (radial and linear EUS)	bile duct scanning augmentation	CH-EUS for pancreatic tumors, automatic segmentation (U-Net system)	pancreatobiliary segmentation and station recognition system (BP MASTER)
Study population	39 patients (randomized to EUS FNA with or without CH-EUS MASTERuse)	4530 images of pancreatic masses; 8 trainees	12 patients, 3575 images (143 sets of 25 frames each)	8113 still images from EUS videos	13,210 images (10,681 for station classification model, 2529 for segmentation model), 781 videos clips (264, 517); 12 trainees	100 patients	4155 images (19,486 for station classification model, 2207 for segmentation model); 8 trainees
Main results	AI for diagnosing pancreatic masses Ac, Sn, Sp, PPV, NPV: 93.8%, 90.9%, 100%, 100%, 83.3%, AI AUC vs. endoscopists 0.923 vs. 0.865 (*p* < 0.05); AI guided EUS-FNA Ac, Sn, Sp, PPV, NPV: 93.8%, 90.9%, 100%, 100%, 83.3%; AI AUC 0.955 vs. control 0.933 (*p* > 0.05)	AI real-time capture and segmentation: dice coefficient of 0.763, recall rate 0.941, precision rate 0.642; Ac 0.842. Median IoU of all cases 0.731. Average IoU of trainees: from 0.80 to 0.87 (*p* = 0.002), average time for identifying lesions (pancreatic body/tail 22.75 vs. 17.98 s *p* < 0.01; pancreatic head/uncinate 34.21 vs. 25.92 s *p* < 0.01).	AI prediction Ac 76% at image level on a dataset with 5 different landmarks	Radial model: mAP 69.67%, F1-score 92%, average IoU (overall between model prediction and expert marking) 79.08%, loss of 0.13. Linear model: mAP 83.43%, F1-score 89%, average IoU 73.48%, total loss 0.16.	AI external validation: Ac in station classification 83.9%, in video set 90.1%. AI segmentation: dice 0.77 in image set; Sn and Sp 89.48% and 82.3% in video set. Trainees aided by AI: Ac in station recognition 76.3% from 60.8% (*p* < 0.01).	concordance rate using IoU 0.77	AI external validation: Ac in station classification 82.4%; dice in segmentation 0.715. AI station classification model: per-frame Ac 86.2% in videos. Trainees aided by the AI: Ac in station recognition 78.4% from 67.2% (*p* < 0.01)

Abbreviations: AI (Artificial Intelligence), CNN (Convolutional Neural Network), CH-EUS (Contrast-Enhanced Endoscopic Ultrasound), EUS (Endoscopic Ultrasound), FNA (Fine Needle Aspiration), Ac (Accuracy), Sn (Sensitivity), Sp (Specificity), PPV (Positive Predictive Value), NPV (Negative Predictive Value), AUC (Area Under the Curve), IoU (Intersection over Union), mAP (mean Average Precision).

## Data Availability

No new data were created or are available for this study. The analysis and conclusions presented in this paper are based on previously published data and existing information.

## Abbreviations

EUS: endoscopic ultrasound; AI: artificial intelligence; ANN: artificial neural network; CNN: convolutional neural network; GMM: Gaussian mixture model; SVM: support-vector machines; SEL: subepithelial lesion; PDAC: pancreatic adenocarcinoma; AIP: autoimmune pancreatitis; CP: chronic pancreatitis; NP: normal pancreas; FLL: focal liver lesions; NET: neuroendocrine tumor; GIST: gastrointestinal stromal tumor; IPMN: intraductal papillary mucinous neoplasia; Ac: accuracy; Sp: specificity; Sn: sensitivity; PPV: positive predictive value; NPV: negative predictive value; TIC: time-intensity curve; AUROC: area under the receiver operating characteristic; AUC: area under the curve; IoU: intersection over union; mAP: mean average precision; FNA: fine needle aspiration; FNB: fine needle biopsy; ROSE: rapid on-site evaluation; MOSE: macroscopic on-site evaluation.

## References

[1] Friedberg S.R., Lachter J. (2017). Endoscopic ultrasound: Current roles and future directions. World J. Gastrointest. Endosc..

[2] Sooklal S., Chahal P. (2020). Endoscopic Ultrasound. Surg. Clin. N. Am..

[3] Liu E., Bhutani M.S., Sun S. (2021). Artificial intelligence: The new wave of innovation in EUS. Endosc. Ultrasound.

[4] Yu K.-H., Beam A.L., Kohane I.S. (2018). Artificial intelligence in healthcare. Nat. Biomed. Eng..

[5] Yamashita R., Nishio M., Do R.K.G., Togashi K. (2018). Convolutional neural networks: An overview and application in radiology. Insights Imaging.

[6] Erickson B.J., Korfiatis P., Akkus Z., Kline T.L. (2017). Machine Learning for Medical Imaging. Radiographics.

[7] Iwasa Y., Iwashita T., Takeuchi Y., Ichikawa H., Mita N., Uemura S., Shimizu M., Kuo Y.-T., Wang H.-P., Hara T. (2021). Automatic Segmentation of Pancreatic Tumors Using Deep Learning on a Video Image of Contrast-Enhanced Endoscopic Ultrasound. J. Clin. Med..

[8] Sinkala M., Mulder N., Martin D. (2020). Machine Learning and Network Analyses Reveal Disease Subtypes of Pancreatic Cancer and their Molecular Characteristics. Sci. Rep..

[9] Simsek C., Lee L.S. (2022). Machine learning in endoscopic ultrasonography and the pancreas: The new frontier?. Artif. Intell. Gastroenterol..

[10] Bi W.L., Hosny A., Schabath M.B., Giger M.L., Birkbak N.J., Mehrtash A., Allison T., Arnaout O., Abbosh C., Dunn I.F. (2019). Artificial intelligence in cancer imaging: Clinical challenges and applications. CA Cancer J. Clin..

[11] LeCun Y., Bengio Y., Hinton G. (2015). Deep learning. Nature.

[12] Murali N., Kucukkaya A., Petukhova A., Onofrey J., Chapiro J. (2020). Supervised Machine Learning in Oncology: A Clinician’s Guide. Dig. Dis. Interv..

[13] Shao D., Dai Y., Li N., Cao X., Zhao W., Cheng L., Rong Z., Huang L., Wang Y., Zhao J. (2022). Artificial intelligence in clinical research of cancers. Brief. Bioinform..

[14] Dumitrescu E.A., Ungureanu B.S., Cazacu I.M., Florescu L.M., Streba L., Croitoru V.M., Sur D., Croitoru A., Turcu-Stiolica A., Lungulescu C.V. (2022). Diagnostic Value of Artificial Intelligence-Assisted Endoscopic Ultrasound for Pancreatic Cancer: A Systematic Review and Meta-Analysis. Diagn. Basel Switz..

[15] Muhammad W., Hart G.R., Nartowt B., Farrell J.J., Johung K., Liang Y., Deng J. (2019). Pancreatic Cancer Prediction Through an Artificial Neural Network. Front. Artif. Intell..

[16] Corral J.E., Hussein S., Kandel P., Bolan C.W., Bagci U., Wallace M.B. (2019). Deep Learning to Classify Intraductal Papillary Mucinous Neoplasms Using Magnetic Resonance Imaging. Pancreas.

[17] Glenny R.W., Robertson H.T., Yamashiro S., Bassingthwaighte J.B. (1991). Applications of fractal analysis to physiology. J. Appl. Physiol..

[18] Carrara S., Di Leo M., Grizzi F., Correale L., Rahal D., Anderloni A., Auriemma F., Fugazza A., Preatoni P., Maselli R. (2018). EUS elastography (strain ratio) and fractal-based quantitative analysis for the diagnosis of solid pancreatic lesions. Gastrointest. Endosc..

[19] Hirai K., Kuwahara T., Furukawa K., Kakushima N., Furune S., Yamamoto H., Marukawa T., Asai H., Matsui K., Sasaki Y. (2022). Artificial intelligence-based diagnosis of upper gastrointestinal subepithelial lesions on endoscopic ultrasonography images. Gastric Cancer.

[20] Ye X.H., Zhao L.L., Wang L. (2022). Diagnostic accuracy of endoscopic ultrasound with artificial intelligence for gastrointestinal stromal tumors: A meta-analysis. J. Dig. Dis..

[21] Ishikawa T., Hayakawa M., Suzuki H., Ohno E., Mizutani Y., Iida T., Fujishiro M., Kawashima H., Hotta K. (2022). Development of a Novel Evaluation Method for Endoscopic Ultrasound-Guided Fine-Needle Biopsy in Pancreatic Diseases Using Artificial Intelligence. Diagnostics.

[22] Parasher G., Wong M., Rawat M. (2020). Evolving role of artificial intelligence in gastrointestinal endoscopy. World J. Gastroenterol..

[23] Rodriguez J.P.M., Rodriguez R., Silva V.W.K., Kitamura F.C., Corradi G.C.A., de Marchi A.C.B., Rieder R. (2022). Artificial intelligence as a tool for diagnosis in digital pathology whole slide images: A systematic review. J. Pathol. Inform..

[24] Marya N.B., Powers P.D., Chari S.T., Gleeson F.C., Leggett C.L., Abu Dayyeh B.K., Chandrasekhara V., Iyer P.G., Majumder S., Pearson R.K. (2021). Utilisation of artificial intelligence for the development of an EUS-convolutional neural network model trained to enhance the diagnosis of autoimmune pancreatitis. Gut.

[25] Marya N.B., Powers P.D., Fujii-Lau L., Abu Dayyeh B.K., Gleeson F.C., Chen S., Long Z., Hough D.M., Chandrasekhara V., Iyer P.G. (2021). Application of artificial intelligence using a novel EUS-based convolutional neural network model to identify and distinguish benign and malignant hepatic masses. Gastrointest. Endosc..

[26] Oh C.K., Kim T., Cho Y.K., Cheung D.Y., Lee B.-I., Cho Y.-S., Kim J.I., Choi M.-G., Lee H.H., Lee S. (2021). Convolutional neural network-based object detection model to identify gastrointestinal stromal tumors in endoscopic ultrasound images. J. Gastroenterol. Hepatol..

[27] Zhang M.-M., Yang H., Jin Z.-D., Yu J.-G., Cai Z.-Y., Li Z.-S. (2010). Differential diagnosis of pancreatic cancer from normal tissue with digital imaging processing and pattern recognition based on a support vector machine of EUS images. Gastrointest. Endosc..

[28] Cazacu I.M., Udristoiu A., Gruionu L.G., Iacob A., Gruionu G., Saftoiu A. (2019). Artificial intelligence in pancreatic cancer: Toward precision diagnosis. Endosc. Ultrasound.

[29] Săftoiu A., Vilmann P., Dietrich C.F., Iglesias-Garcia J., Hocke M., Seicean A., Ignee A., Hassan H., Streba C.T., Ioncică A.M. (2015). Quantitative contrast-enhanced harmonic EUS in differential diagnosis of focal pancreatic masses (with videos). Gastrointest. Endosc..

[30] Echle A., Rindtorff N.T., Brinker T.J., Luedde T., Pearson A.T., Kather J.N. (2021). Deep learning in cancer pathology: A new generation of clinical biomarkers. Br. J. Cancer.

[31] Kurita Y., Kuwahara T., Hara K., Mizuno N., Okuno N., Matsumoto S., Obata M., Koda H., Tajika M., Shimizu Y. (2019). Diagnostic ability of artificial intelligence using deep learning analysis of cyst fluid in differentiating malignant from benign pancreatic cystic lesions. Sci. Rep..

[32] Hashimoto Y., Ohno I., Imaoka H., Takahashi H., Mitsunaga S., Sasaki M., Kimura G., Suzuki Y., Watanabe K., Umemoto K. (2018). Mo1296 Reliminary result of computer aided diagnosis (cad) performance using deep learning in eus-fna cytology of pancreatic cancer. Gastrointest. Endosc..

[33] Inoue H., Ogo K., Tabuchi M., Yamane N., Oka H. An automatic visual inspection method based on supervised machine learning for rapid on-site evaluation in EUS-FNA. Proceedings of the 2014 Proceedings of the SICE Annual Conference (SICE).

[34] Bencze J., Szarka M., Kóti B., Seo W., Hortobágyi T.G., Bencs V., Módis L.V., Hortobágyi T. (2021). Comparison of Semi-Quantitative Scoring and Artificial Intelligence Aided Digital Image Analysis of Chromogenic Immunohistochemistry. Biomolecules.

[35] Meyerholz D.K., Beck A.P. (2018). Fundamental Concepts for Semiquantitative Tissue Scoring in Translational Research. ILAR J..

[36] Baxi V., Edwards R., Montalto M., Saha S. (2022). Digital pathology and artificial intelligence in translational medicine and clinical practice. Mod. Pathol..

[37] Bera K., Schalper K.A., Rimm D.L., Velcheti V., Madabhushi A. (2019). Artificial intelligence in digital pathology—New tools for diagnosis and precision oncology. Nat. Rev. Clin. Oncol..

[38] Johnson G., Webster G., Boškoski I., Campos S., Gölder S.K., Schlag C., Anderloni A., Arnelo U., Badaoui A., Bekkali N. (2021). Curriculum for ERCP and endoscopic ultrasound training in Europe: European Society of Gastrointestinal Endoscopy (ESGE) Position Statement. Endoscopy.

[39] Kuwahara T., Hara K., Mizuno N., Haba S., Okuno N., Koda H., Miyano A., Fumihara D. (2021). Current status of artificial intelligence analysis for endoscopic ultrasonography. Dig. Endosc..

[40] Tang A., Tian L., Gao K., Liu R., Hu S., Liu J., Xu J., Fu T., Zhang Z., Wang W. (2023). Contrast-enhanced harmonic endoscopic ultrasound (CH-EUS) MASTER: A novel deep learning-based system in pancreatic mass diagnosis. Cancer Med..

[41] Tang A., Gong P., Fang N., Ye M., Hu S., Liu J., Wang W., Gao K., Wang X., Tian L. (2023). Endoscopic ultrasound diagnosis system based on deep learning in images capture and segmentation training of solid pancreatic masses. Med. Phys..

[42] Bonmati E., Hu Y., Grimwood A., Johnson G.J., Goodchild G., Keane M.G., Gurusamy K., Davidson B., Clarkson M.J., Pereira S.P. (2022). Voice-Assisted Image Labeling for Endoscopic Ultrasound Classification Using Neural Networks. IEEE Trans. Med. Imaging.

[43] Robles-Medranda C., Oleas R., Valle R.S.D., Mendez J.C., Alcívar-Vásquez J.M., Puga-Tejada M., Lukashok H.P. (2021). Application of artificial intelligence for real-time anatomical recognition during endoscopic ultrasound evaluation: A pilot study. Gastrointest. Endosc..

[44] Yao L., Zhang J., Liu J., Zhu L., Ding X., Chen D., Wu H., Lu Z., Zhou W., Zhang L. (2021). A deep learning-based system for bile duct annotation and station recognition in linear endoscopic ultrasound. EBioMedicine.

[45] Zhang J., Zhu L., Yao L., Ding X., Chen D., Wu H., Lu Z., Zhou W., Zhang L., An P. (2020). Deep learning-based pancreas segmentation and station recognition system in EUS: Development and validation of a useful training tool (with video). Gastrointest. Endosc..

[46] Khan R., Plahouras J., Johnston B.C., Scaffidi M.A., Grover S.C., Walsh C.M. (2019). Virtual reality simulation training in endoscopy: A Cochrane review and meta-analysis. Endoscopy.

[47] Spadaccini M., Koleth G., Emmanuel J., Khalaf K., Facciorusso A., Grizzi F., Hassan C., Colombo M., Mangiavillano B., Fugazza A. (2022). Enhanced endoscopic ultrasound imaging for pancreatic lesions: The road to artificial intelligence. World J. Gastroenterol..

